# Regional biomechanical characterization of the spinal cord tissue: dynamic mechanical response

**DOI:** 10.3389/fbioe.2024.1439323

**Published:** 2024-08-16

**Authors:** Chen Jin, Jiang-ming Yu, Ran Li, Xiao-jian Ye

**Affiliations:** ^1^ Laboratory of Key Technology and Materials in Minimally Invasive Spine Surgery, Tongren Hospital, Shanghai Jiao Tong University School of Medicine, Shanghai, China; ^2^ Center for Spinal Minimally Invasive Research, Shanghai Jiao Tong University, Shanghai, China; ^3^ Department of Orthopaedics, Tongren Hospital, Shanghai Jiao Tong University School of Medicine, Shanghai, China; ^4^ Department of Endocrinology, Shanghai East Hospital, Tongji University School of Medicine, Shanghai, China

**Keywords:** spinal cord mechanics, dynamic mechanical response, viscoelasticity, mechanical properties, indentation

## Abstract

Characterizing the dynamic mechanical properties of spinal cord tissue is deemed important for developing a comprehensive knowledge of the mechanisms underlying spinal cord injury. However, complex viscoelastic properties are vastly underexplored due to the spinal cord shows heterogeneous properties. To investigate regional differences in the biomechanical properties of spinal cord, we provide a mechanical characterization method (i.e., dynamic mechanical analysis) that facilitates robust measurement of spinal cord *ex vivo*, at small deformations, in the dynamic regimes. Load-unload cycles were applied to the tissue surface at sinusoidal frequencies of 0.05, 0.10, 0.50 and 1.00 Hz *ex vivo* within 2 h *post mortem*. We report the main response features (e.g., nonlinearities, rate dependencies, hysteresis and conditioning) of spinal cord tissue dependent on anatomical origin, and quantify the viscoelastic properties through the measurement of peak force, moduli, and hysteresis and energy loss. For all three anatomical areas (cervical, thoracic, and lumbar spinal cord tissues), the compound, storage, and loss moduli responded similarly to increasing strain rates. Notably, the complex modulus values of *ex vivo* spinal cord tissue rose nonlinearly with rising test frequency. Additionally, at every strain rate, it was shown that the tissue in the thoracic spinal cord was significantly more rigid than the tissue in the cervical or lumbar spinal cord, with compound modulus values roughly 1.5-times that of the lumbar region. At strain rates between 0.05 and 0.50 Hz, tan δ values for thoracic (that is, 0.26, 0.25, 0.06, respectively) and lumbar (that is, 0.27, 0.25, 0.07, respectively) spinal cord regions were similar, respectively, which were higher than cervical (that is, 0.21, 0.21, 0.04, respectively) region. The conditioning effects tend to be greater at relative higher deformation rates. Interestingly, no marked difference of conditioning ratios is observed among all three anatomical regions, regardless of loading rate. These findings lay a foundation for further comparison between healthy and diseased spinal cord to the future development of spinal cord scaffold and helps to advance our knowledge of neuroscience.

## 1 Introduction

The spinal cord relays information between the brain and the periphery, controls voluntary movements of the muscles in the trunk and extremities, and receives sensory information from the body. Traumatic spinal cord injury (SCI) often results in serious reduction or loss of sensory, motor, and autonomic functions below the injury site (i.e., cervical, thoracic and lumbar segments). The incidence of traumatic SCI ranges from 10.4 to 83.0 per million people each year in different countries and regions (Cheriyan et al., 2014; Singh et al., 2014; Hu et al., 2023). Accidents on the road, falls, acts of violence, and physical injuries in sports are common causes due to high speed and dynamic events (Devivo, 2012; Cheriyan et al., 2014; Fitzharris et al., 2014; Singh et al., 2014; Hu et al., 2023). Serious SCI represents a significant physical, psychological and financial burden for patients and their families. Despite this, effective treatment strategies remain absent.

Regenerative medicine has recently begun to offer new paradigms in the treatment of the central nervous system (CNS) disorders, with many regenerative strategies underpinned by biomaterial-based approaches (Führmann and Shoichet, 2018). Tissue-engineered spinal cord transplants have been used to mimic the anatomical structure and approximate function of native spinal cord tissues, provide essential structural or mechanical support (Ahuja et al., 2017; Koffler et al., 2019). However, the strategies to engineer the tissue structures of the spinal cord are challenging due to the architectural and functional complexity (Liu et al., 2018; Shao et al., 2019). Mechanical properties are an essential consideration for any biomaterial implanted into the spinal cord tissue. At the macroscopic level, any undue forces occurring at the biomaterial-tissue interface will disrupt structural continuity and hinder traversing cells or axons (Engler et al., 2006; Hadden et al., 2017). At the microscopic level, previous studies have revealed that stiffness of the local CNS tissue microenvironment has been implicated in a range of cell behaviours, including causing astrocytes cell hypertrophy (Moshayedi et al., 2010; Pekny and Pekna, 2014), altering the differentiation, adhesion and migration of therapeutic stem cells (Moshayedi et al., 2014). Characterization the mechanical properties of spinal cord tissue is, therefore, critical for determining the effectiveness of biomaterial-based regenerative medicine strategies.

Currently, several experiments have explored the bulk mechanical characteristics of spinal cord tissue at the macro-level (tissue level) (Fiford and Bilston, 2005; Clarke et al., 2009; Saxena et al., 2009; Shetye et al., 2014; Karimi et al., 2017; Ramo et al., 2018a; Bartlett et al., 2020; Nishida et al., 2020). Most biomechanical studies were conducted *ex vivo*, although a few *in vivo* measures were also recorded (Ramo et al., 2018b). However, robustly quantifying the mechanical properties remains acutely a daunting challenge, because, from a biomechanical standpoint, the spinal cord is a highly complex and supersoft organ. It composes of white matter, gray matter, cerebrospinal fluid, meningeal tissues (i.e., the spinal dura mater, arachnoid mater, and the pia mater) and surrounding vasculature with somewhat distinct mechanical characteristics (Ramo et al., 2018a). Furthermore, it should be noted that different segments of spinal cord may present with various mechanical properties. A recent study by Dai et al. (Tang et al., 2022) revealed that among patients with complete SCI, improvements in motor function occurred in patients with cervical but not thoracic injury, which indicted that different injury site might lead to varying degrees of paralysis when suffered from similar external shocks. A possible reason was the difference of spinal cord unique regional mechanical properties. It is known that spinal cord white matter mainly consists of glial cells and long, myelinated, highly orientated axons extending along the craniocaudal (i.e., head-to-tail) axis, connecting the brain to the rest of the body. As a whole, gray and white matter behave like an isotropic and transversely isotropic material, respectively. Cell distribution and axon orientation determine the mechanical behavior of local spinal cord tissues (Koser et al., 2015). To date, regional differences regarding the dynamic characteristic features of *ex vivo* spinal cord (hysteretic behavior, rate dependence and nonlinearity) have not been adequately investigated, and this knowledge is essential for a better understand of the local mechanobiological processes that influence the mechanics of SCI.

Due to a significant degree of heterogeneity in experimental techniques, like involving various species of the subjects (non-human primate, porcine, ewe or rat), *post mortem* time (tens of minutes or hours), the loading configurations (compression, tension or shear), sample size (small or large), and loading modes (stress relaxation or creep), it might be challenging to reconcile the measurement data of different research from different labs (Cheng et al., 2008; McKee et al., 2011; Bartlett et al., 2016). For accurately characterizing the time-dependent mechanical response of biological soft tissue, indentation is a validated technique. Despite the fact that the tissue response was extensively defined *ex vivo* under numerous reference modes of deformation (i.e., compression (Sparrey and Keaveny, 2011; Karimi et al., 2017; Jannesar et al., 2018), tension (Tunturi, 1978; Oakland et al., 2006; Luna et al., 2013), or shear (Bilston et al., 1997; Bilston et al., 2001)) these methods require reasonably large samples, preventing the classification of similar samples of limited anatomic locations depending on the complicated tissue structure detected by histology. On the other hand, indentation examination is well-suited for evaluating the mechanical characteristics of spinal cord tissue on the same spatial scale as anatomical regions. It is unnecessary to prepare homogeneous specimens because the probe may be simply positioned in the interest area.

In an attempt to completely analyze and contrast the nonlinear dynamic indentation responses of local spinal cord tissue, this study includes a comprehensive set of experimental data performed *ex vivo* on the cervical, thoracic, and lumbar regions. Measurements of elasticity and energetics, consisting of peak force, moduli, and hysteresis and energy loss, are presented. Additionally, the effects of dura mater, preconditioning and rates are further explored and cross-examined to divulge directional and regional dependencies. To accomplish the work, we exploited the advantages offered by dynamic mechanical analysis. The resulting quantitative set of mechanical measures presents a basic experimental database that may fill the knowledge void which continues to hinder the development of constitutive models and spinal cord scaffold designs, and lay a foundation for future comparisons to investigate differences between healthy and diseased spinal cord.

## 2 Materials and methods

### 2.1 Tissue harvest and specimen preparation

The UK Animals (Scientific Procedures) Act of 1986 was followed for all experimental protocol animal related. The Animal Welfare Committee of Tongren Hospital affiliated to Shanghai Jiaotong University School of Medicine in Shanghai, China, gave its approval to the protocols for animal experiments.

This study comprised two distinct phases. The first (Singh et al., 2014) was an exploratory test phase, wherein the initial mechanical information regarding the *ex-vivo* spinal cord tissue (cervical, thoracic and lumbar) was gathered and characterised. In this phase, a total of 10 rats were used to investigate the effects of dura mater via a series of cyclic loading tests at 0.05, 0.10, 0.50 and 1.00 Hz. The second (Cheriyan et al., 2014) comprised a full-scale test phase wherein the tested procedures were performed to systematically explore the dynamic viscoelastic properties of regional spinal cord tissue.

Adult female Sprague-Dawley rats (11–12 weeks, 250 ± 10 g) were utilized. To induce death in the rats, intraperitoneal pentobarbitone sodium (100 mg/kg) was injected. Cardiac perfusion with ice-cold phosphate-buffered saline (PBS, pH 7.4) then confirmed the stoppage of circulation permanently. Perforations were formed in the thoracolumbar tissue on both sides of the spinal column, and two para-spinal pores were created by bluntly slicing the tissue. Following this step, the ribs, surrounding muscles, and connective tissue were all dissected apart in order to expose the spinal column. Between the base of the head and above the sacrum, transverse incisions were conducted to remove the full spinal column. The spinal cord was then smoothly withdrawn from the spinal canal through a syringe expulsion technique (Kennedy et al., 2013). We next delicately removed any leftover connective tissue or overlaying dura using thin forceps. The harvested spinal cord tissue immediately soaked in PBS at 4°C Celsius. The *ex vivo* spinal cord was then placed in a culture dish of 100 mm in diameter, positioned with the ventral side facing up, and adhered to the slide using cyanoacrylate adhesive. To avoid tissue degeneration, chilled PBS was used to hydrate the material. Therefore, the culture dish was put beneath the indenter tip, and the sample was allowed to acclimate for 10 min previous to indentation testing. Due to the fact that the mechanical properties of biological tissues are susceptible to substantial transformation during the *postmortem* period, indentation studies were conducted within 2 h of the rats’ deaths at room temperature (24°C) (Saxena et al., 2009). Separating intermingled gray and white matter of the spina cord tissue was not appropriate for this study was due to the fact that the separation/cutting process required additional changes to the virgin state of the tissue (by shearing or stretching) that were supposed to be too costly in relation to the benefit gained.

### 2.2 Test apparatus

**FIGURE 1:**
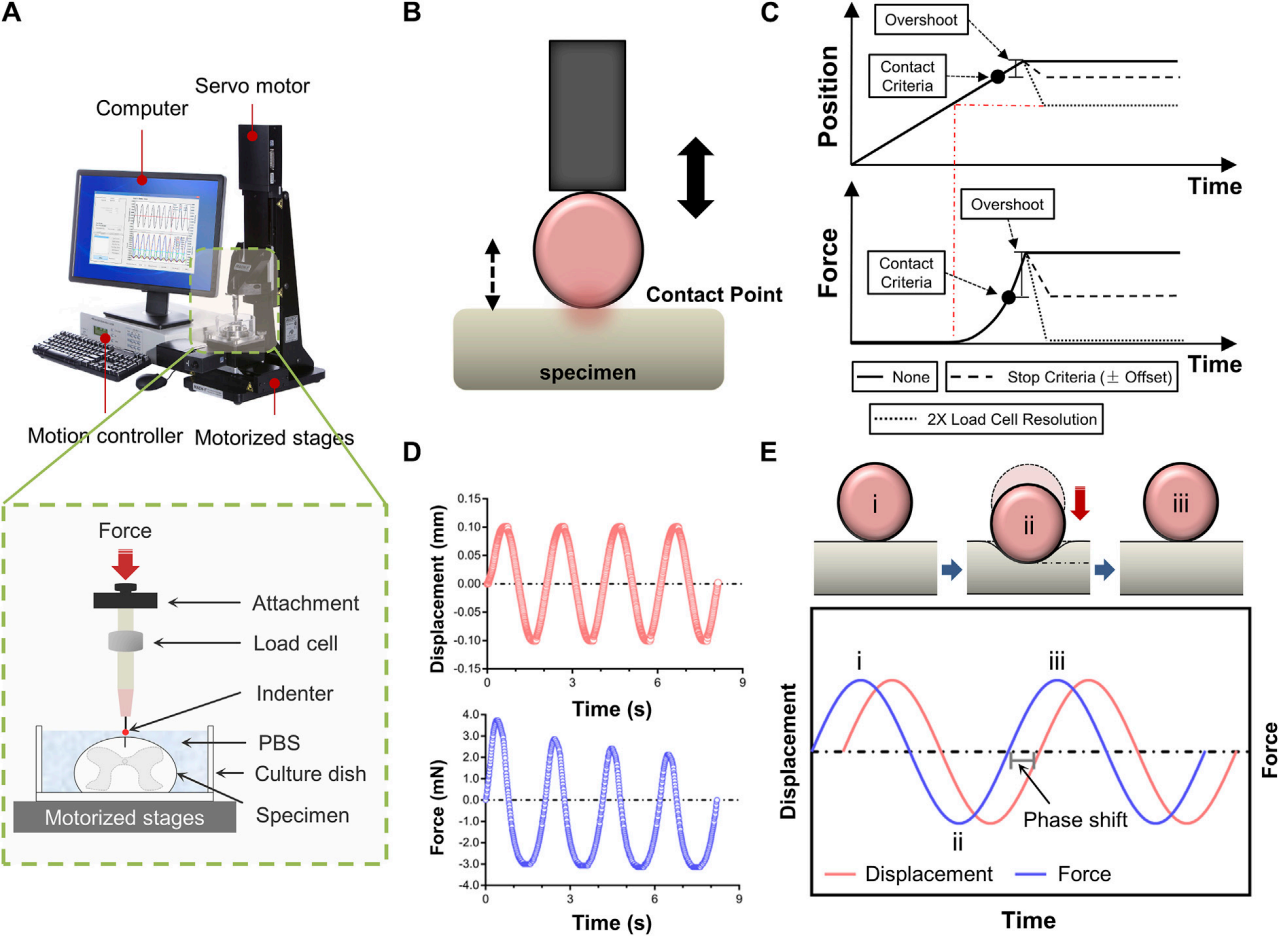
Experimental setup for tissue indentation testing. **(A)** Test apparatus shows indentation testing rig and assistive devices. Mechanical indentation was conducted by a Mach-1 Model V500css test device. The green dashed rectangle depicts schematic of indentation testing setup. **(B)** Schematic of indenter tip position relative to specimen surface during contact determination procedure *ex vivo*. **(C)** Contact criteria. Depending on the predefined stage velocity, the stage will continue to move (position will overshoot), more or less, following the reaching of the stop criteria (hardware delay). In order to compensate for this overshoot, this function automatically analyses the force-displacement curve generated and repositions the stage based on the selected stage repositioning option. “None”, the stage remains at the position where it has stopped (overshoot). “Stop Criteria”, the stage is repositioned where the load variation first corresponded to the stop criteria (overshoot compensation). “2X Load Resolution”, the stage is repositioned where the load variation first corresponded to twice the load cell resolution (surface detection). **(D)** Schematic representation of force-displacement and force-time curves recorded by dynamic mechanical indentation. **(E)** Overview of the dynamic mechanical indentation. Schematic representation of how samples were compressed at different test frequencies using an oscilatory dynamic mechanical analysis method (the upper diagram). Schematic representation of the complex information gained from oscilatory testing, including damping and phase lag, which allows measurement of compound, storage and loss moduli and tan delta. Circled numbers in the lower diagram correspond to the phase of indentation in the upper diagram.

Figure 1A depicted a schematic of how indentation tests on spinal cord tissue are set up. Mechanical characteristics were evaluated *ex vivo* using a Mach-1 Model V500css Device (Biomomentum Inc., Laval, QC, Canada). A single-axial load cell recorded normal load while precisely measuring surface orientation at each place (1.50 N range and 0.07 mN resolution on the vertical axis). The Mach-1 Mechanical Testing System consisted of the tester frame, three motorized stages, a motion controller, a load cell, a load cell amplifier, a computer, and testing chambers and fittings were available as upgrades. In addition to supplying the load cell with electricity, the amplifier performed the additional function of converting the signal representing the measured force into a digital value and sending it to the computer. Mach-1 Motion was the software that ran behind the motion controller, which in turn controlled the stage.

### 2.3 Indentation testing

Indentation tests were done at three anatomical regions (i.e., cervical, thoracic and lumbar spinal cord tissues), respectively. The indenter had a spherical tip with a 0.50 mm diameter. Specimens of the spinal cord were put beneath the indenter tip, and the load and displacement values were set to zero. When using a spherical indenter tip to evaluate a nonlinear specimen response, accurately and repeatedly identifying the contact point between the tip and the sample is particularly crucial. Hence, we developed a more systematic determination technique to prevent misidentification and account for low force variations. The contact function moved a stage until a predefined load variation (stop criteria) was measured by the load cell. Then, the force-displacement curve was analysed, and the stage was repositioned based on the selected stage repositioning option. Depending on the predefined stage velocity, the stage continued to move (position will overshoot) till the stop criteria was achieved (hardware delay). To compensate for this overshoot, the function automatically analysed the generated force-displacement curve and repositioned the stage based on the selected stage repositioning option (Figures 1B, C). Each indentation sequence initiated with an adjustment to the vertical z-position of the indenter, as determined by the motion controller. This technique involved lightly “tapping” the surface of the specimen at a speed of 0.05 mm/s. A 10% pre-strain ramp was applied initially to assure sample contact. Test sequences comprised of 3-5 cycles of dynamic mechanical analysis to an amplitude of 0.10 mm (3% strain) at sinusoidal ascending rates of 0.05, 0.10, 0.50 or 1.00 Hz (Figures 1D, E). Dynamic mechanical analysis was used to evaluate elastic and viscous portions of the spinal cord behavior. Dynamic mechanical properties are usually expressed in terms of compound dynamic modulus (M) with storage (M′) and loss modulus (M″) components according to Equation 1. The storage modulus (M′) can be associated with the elastic behavior that the tissue returns to its original shape when the external stress is removed. The loss modulus (M″) represents the viscous behavior that the tissue stops deforming and does not return to its original shape when the external stress is removed. Both M′ and M″ were also used to calculate tan delta (δ) according to Equation 2 which indicates the tissue’s viscous (tan δ close to ∞) or elastic behavior (tan δ close to zero). The tan δ is the energy consumption caused by hysteresis in each cycle, also known as internal consumption or mechanical loss, representing the ability of the tissue itself to absorb external energy. At least 5 min were allowed for the specimen to recover between repeated sequences at different locations. Data with a relocation inaccuracy >5% were eliminated. The data was gathered at a sample frequency of 100 Hz.

$$M=M^{\prime }+\text{iM}’’$$ (1)

$$\mathrm{Tan}\;\delta =M^{\prime \prime }/\;M^{\prime }$$ (2)

### 2.4 Statistical analysis

All statistical analysis was performed with SPSS 20.0 (SPSS Inc., Chicago, IL, USA). The Shapiro-Wilk test was used to determine whether or not the data were normally distributed. While, the Student’s two-tailed *t*-test was utilized to determine whether or not there was a significant difference between the two groups. When analyzing several groups, either a one-way ANOVA followed by Tukey’s *post hoc* analysis or a Kruskal–Wallis ANOVA followed by Games-*post* Howell’s *hoc* analysis was used depending on whether or not the variances across the groups were comparable to one another. In the Supplementary Tables, exact *p* values are provided. Statistically significant differences were indicated by *p* values 0.05. The *p* values in the figures are as follows: ^*^ *p* < 0.05, ^**^ *p* < 0.01, and ^***^ *p* < 0.001.

## 3 Results

### 3.1 Preliminary observations: effect of dura mater on spinal cord tissue response

**FIGURE 2:**
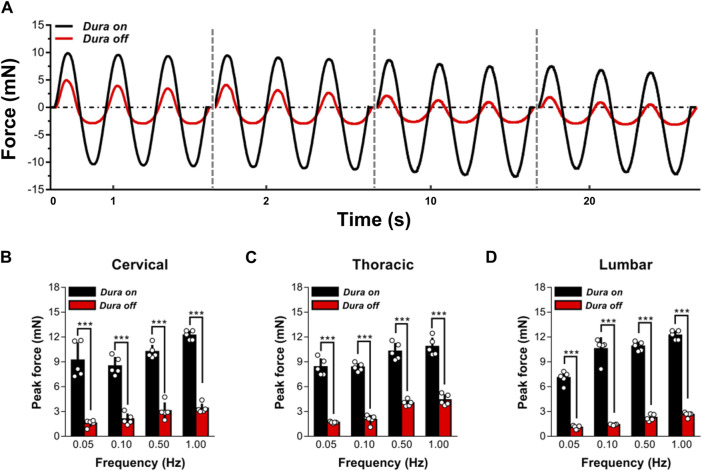
Effect of dura mater on changes to the local mechanical response of *ex vivo* spinal cord tissue. **(A)** Indenter force response shown for two representative sets of measurements performed with or without dura mater. Cyclic loading tests at 0.05, 0.10, 0.50 and 1.00 Hz are shown sequentially on the same time axis. **(B–D)** The quantitative comparisons of the peak force for cervical, thoracic and lumbar tissues with and without dura mater. Error bars represent standard deviation. ^***^ indicates *p* < 0.001. Exact *p* values for **(B–D)** are listed in Supplementary Table S1.

To analyze the mechanical effect of dura mater on *ex vivo* spinal cord tissue response, indentation experiments on *ex vivo* specimens, with (N = 5) or without (N = 5) the dura mater, were performed (Figure 2). Findings indicated that the reaction of dura-free tissue in various spinal segments was much more compliant than that of intact tissue (Figure 2A). It was discovered that intact tissue is significantly stiffer than dura-free tissue, with the peak force values more than twofold at various frequencies ranging from 0.05 to 1.00 Hz (*p* < 0.001; Figures 2B–D; Supplementary Table S1). Considering the pronounced effect of dura mater, all measurements reported hereafter were thus tested on dura-free tissue.

### 3.2 Test repeatability: location dependence, rate order indifference

**FIGURE 3:**
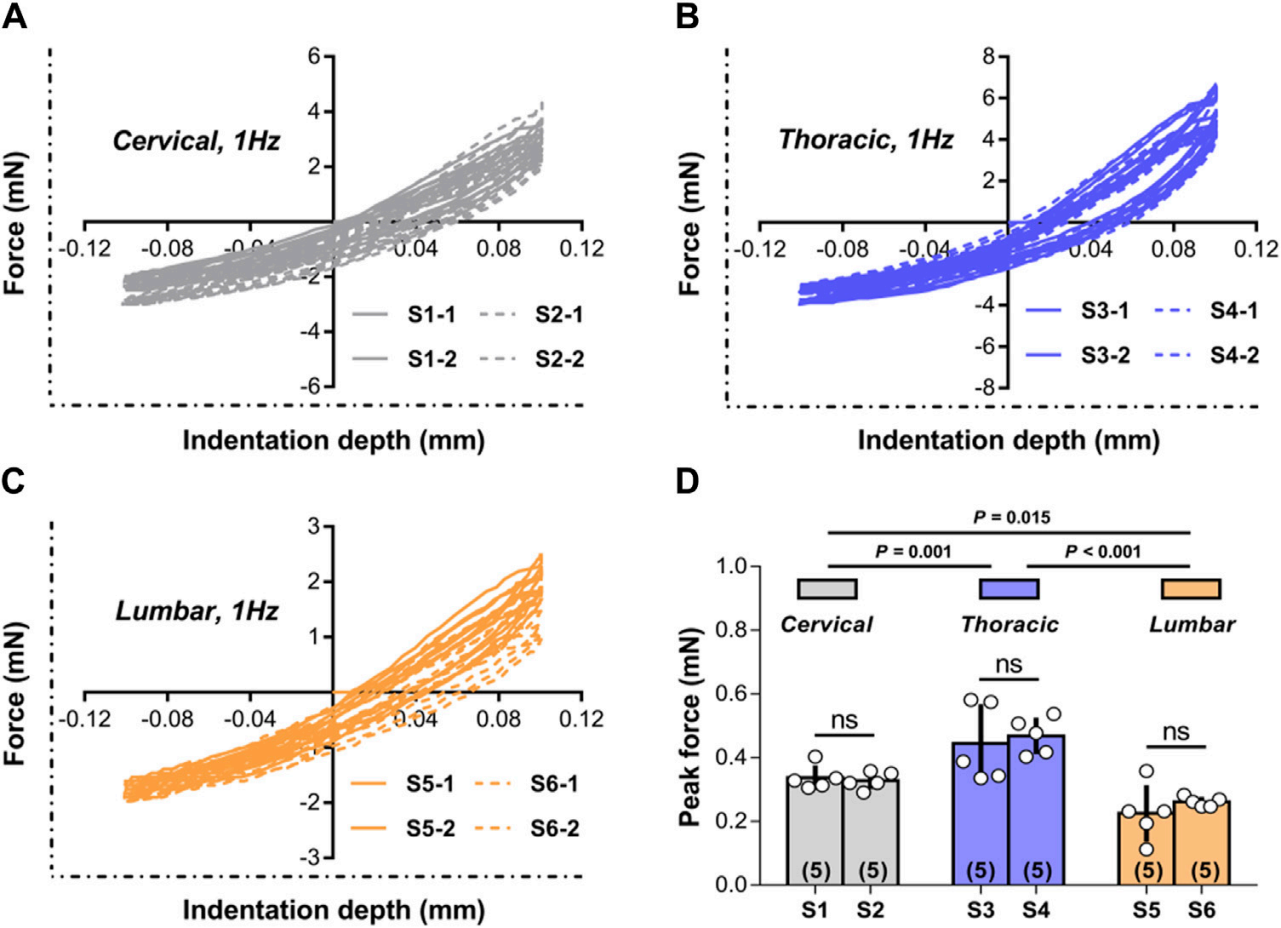
Test repeatability: location dependence. **(A–C)** Representative responses measured *ex vivo* during repeated tests conducted in cervical, thoracic and lumbar spinal cord tissue at the two random indentation sites to 0.10 mm depth at 1.00 Hz frequency. All indentation tests were repeated twice within each site, with distinct test sequences separated by at least 5 min recovery phases. **(D)** Peak forces reached at the end of the first loading ramp for the two indentation sites submitted to 1.00 Hz load-unload cycles *ex vivo* in different spinal cord regions. Error bars represent standard deviation. Numbers in parentheses refers to the number of independent measurements performed on a total of five animals.

Figures 3A–C shows representative responses evaluated during multiple experiments done on cervical, thoracic, and lumbar spinal cord tissue at two random indentation sites at a frequency of 1.00 Hz. The responses to consecutive indentation *ex vivo* were shown to be reliable and reproducible in the cervical, thoracic, and lumbar portions of the spinal cord. Comparing the peak force measures achieved at the completion of the initial (unconditioned) loading ramp to the tissue, there were no statistically significant differences between indentation sites at each spinal cord segment (*p* > 0.05; Figure 3D), but there were noticeable differences across three areas (i.e., cervical, thoracic, and lumbar tissues) (*p* < 0.001; Figure 3D). These findings revealed that the reaction of the tissue was poorly dependent on the place of local indentation, although different anatomical areas did not share similar macroscopic mechanical properties.

**FIGURE 4:**
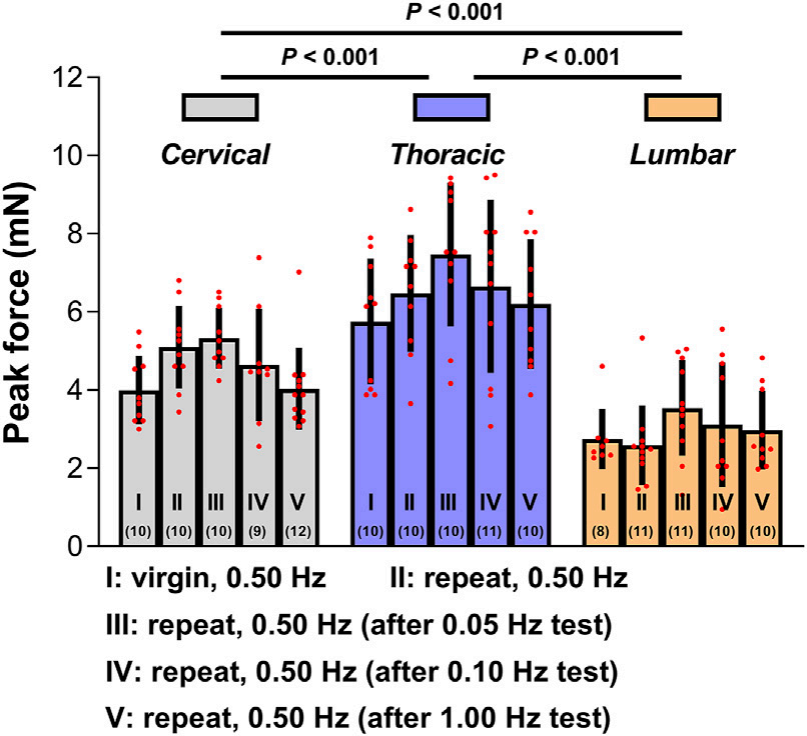
Test repeatability: rate order independence. Peak forces reached at the end of the first loading ramp *ex vivo* under five conditions. Error bars represent standard deviation. Numbers in parentheses correspond to numbers of measurements performed under each condition for a total of six animals. Exact *p* values are listed in Supplementary Table S2.

To further evaluate the quantitative repeatability in the mechanical indentation measurements, we evaluated tissue reactions to 0.50 Hz cyclic indentation (as calculated by the peak forces achieved upon first loading for every sequence of load-unload segments employed on the tissues). These peak forces were contrasted inside and between indentation locations based on five conditions: (I) the indentation site has not been previously tested; (II) the indentation site was subjected to a minimum of one sequence of 4-5 load-unload segments at 0.50 Hz; (III-IV) the indentation site was previously subjected to a minimum of one sequence of 3-5 load-unload segments at relative lower indentation rates (i.e., 0.05 and 0.10 Hz, respectively) without previously been submitted to the applicable greater rate (i.e., 1.00 Hz) indentation sequences; and (V) the indentation site was previously subjected to at least one sequence of 3-5 load-unload segments at relevant a higher indentation rate (i.e., 1.00 Hz) with no previous submission to relative lower rate (i.e., 0.05 Hz and/or 0.10 Hz) indentation sequences. Figure 4 demonstrates the findings. Although the mechanical responses measured on virgin (previously untested) indentation sites in the cervical, thoracic, and lumbar spinal cord segments were significantly softer than those retrieved on previous tested sites, these distinctions were not statistically significant (*p* > 0.05 for all cases compared to the virgin case; Figure 4; Supplementary Table S2). Under the proper premise (i.e., the *ex vivo* tissue hydrated states was preserved, the tissue was left to recover for at least 5 min before every test, and appropriate contact criteria at the start of each test sequence was confirmed), these experimental findings indicated that precompressing the tissue or altering the rate order in the cervical, thoracic, or lumbar segment has no impact significantly on subsequent values.

### 3.3 Comparison of mechanical response of spinal cord tissue

**FIGURE 5:**
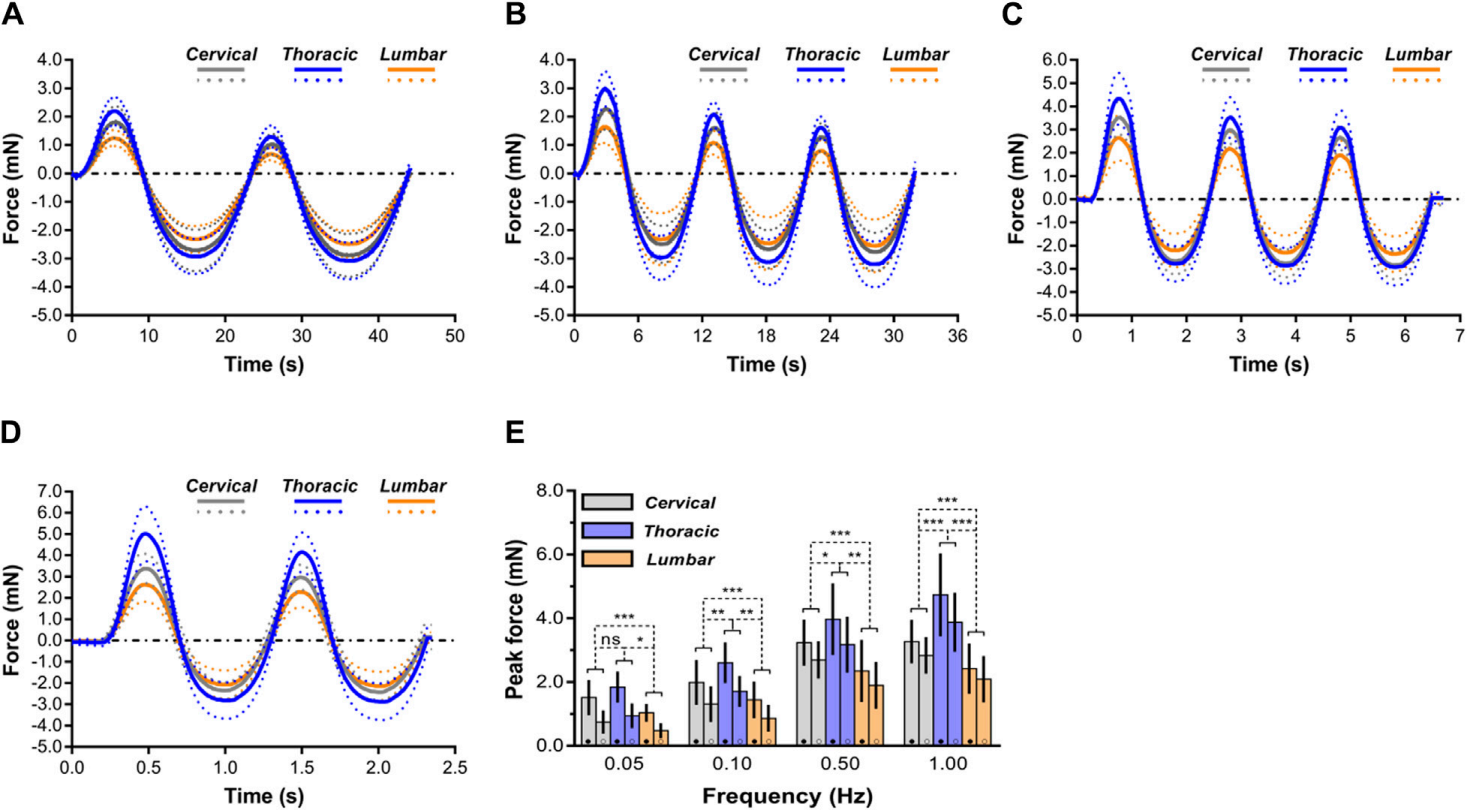
Comparison of mechanical response of spinal cord tissue. **(A–D)** Indentation responses contrasted *ex vivo* in cyclic load-unload at 0.05 Hz **(A)**, 0.10 Hz **(B)**, 0.50 Hz **(C)** and 1.00 Hz **(D)**. Dashed lines correspond to average plus or minus standard deviation. For clarity purposes, only the first two load-unload cycles are shown in the 0.05 and 1.00 Hz cases. **(E)** Comparisons of mechanical responses of three regions (i.e., cervical, thoracic and lumbar spinal cord tissues) in terms of peak forces reached at the end of the first loading ramp (●) and of the second loading ramp (○) for the four different rates of indentation. Error bars represent standard deviation. Exact *p* values are listed in Supplementary Table S3.

Average mechanical responses (with standard deviations) conducted at 0.05, 0.10, 0.50, 1.00 Hz to 0.10 mm indentation displacement *ex vivo* are reported in Figure 5. The thoracic spinal cord tissue behaved significantly less compliant than that of the cervical or lumbar tissue, regardless of loading rates frequency. For further comparison of indentation responses among various regions, we quantitatively analyzed the peak forces values reached upon first loading and second loading (Figure 5E). The results showed that the ‘‘unconditioned’’ response, namely, the indentation response upon first loading, was determined to be significantly larger than that found following cyclic reloading in the large majority of cases (Supplementary Table S3), consistent within prior observations on *ex vivo* or *in vivo* porcine brain specimens tested in uniaxial compression (Prevost et al., 2011a; Prevost et al., 2011b; Kennedy et al., 2013). In addition, as loading rates increased, more pronounced difference was observed among all three anatomical regions (Figure 5E).

### 3.4 Significant non-linear rate dependence of spinal cord tissue

For cervical, thoracic, and lumbar spinal cord tissues, it was determined that indentation rates are significantly rate dependent. According to the peak forces at 0.10 mm penetration depth, these variations were statistically significant (*p* < 0.05; Figure 5; Supplementary Table S3). Interestingly, when displacement rates increased, the relative sensitivity of the reaction to displacement rate decreased. As shown in Figure 5E, *ex vivo* thoracic spinal cord tissue revealed that peak forces at 0.10 Hz surpassed those at 0.05 Hz by a factor of 0.42, whereas peak forces at 1.00 Hz surpassed those at 0.50 Hz by only a factor of 0.19 (Supplementary Table S3).

### 3.5 Comparison of conditioning effects of spinal cord tissue

**FIGURE 6:**
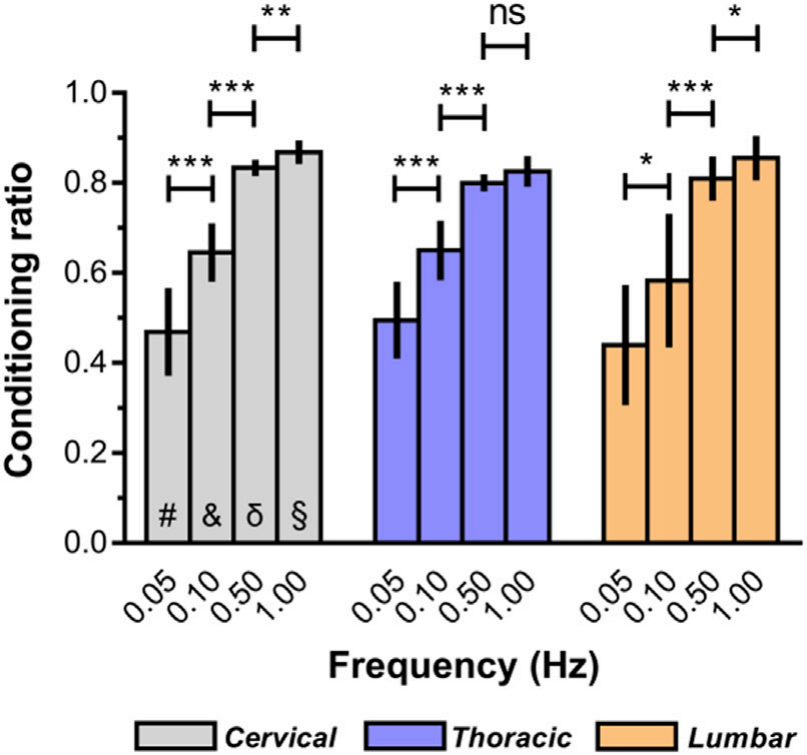
Conditioning ratios for each indentation rate for *ex vivo* spinal cord tissue. The conditioning ratio is defined as the ratio between the peak forces reached at the end of the second loading ramp (conditioned) and the peak forces reached at the end of the first loading ramp (unconditioned). Error bars represent standard deviation. ^*^ indicates *p* < 0.05, ^**^ indicates *p* < 0.01, ^***^ indicates *p* < 0.001. # indicates no significant differences across three regions at 0.05 Hz and indicates no significant differences across three regions at 0.10 Hz δ indicates no significant differences across three regions at 0.50 Hz § indicates no significant differences across three regions at 1.00 Hz. Exact *p* values are listed in Supplementary Tables S4, S5.

The macroscopic indentation response revealed a high degree of “conditioning” among the mechanical properties of *ex vivo* spinal cord tissue. As illustrated in Figure 6, the conditioning effects tended to be greater at relative higher deformation rates (Supplementary Table S4). For instance, *ex vivo* conditioning ratios averaged 0.87 ± 0.26 at 1.00 Hz for cervical spinal cord, compared with 0.47 ± 0.10 at lower rates (0.05 Hz). Interestingly, we also found that no marked difference of conditioning ratios was observed among all three anatomical regions, regardless of loading rate (Figure 6; Supplementary Table S5).

### 3.6 Local mechanical properties of spinal cord tissue

**FIGURE 7:**
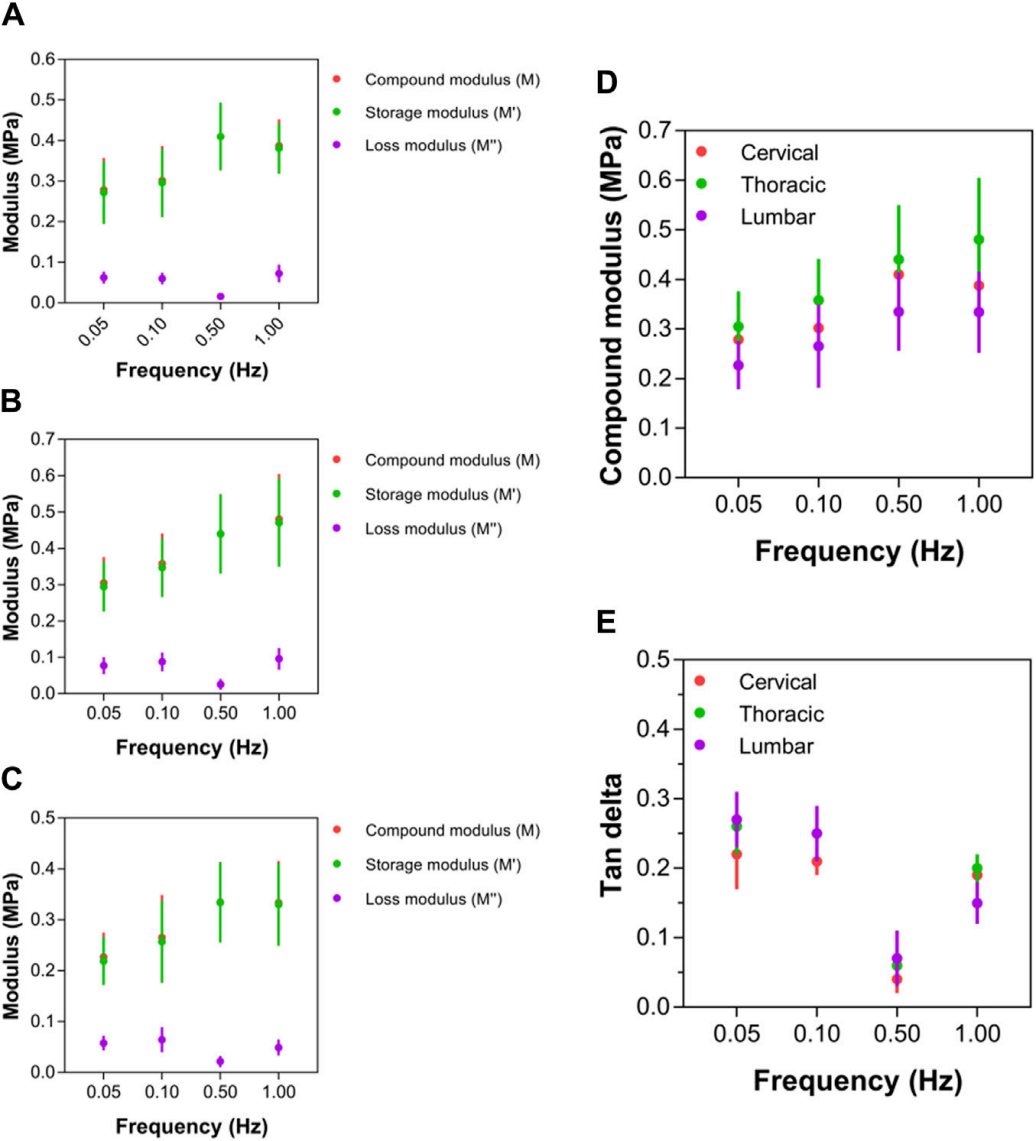
Local mechanical properties of cervical, thoracic and lumbar spinal cord tissue. **(A–C)** Compound, storage and loss modulus values for cervical, thoracic and lumbar spinal cord, respectively. **(D)** Comparison of compound modulus values for cervical, thoracic and lumbar spinal cord. **(E)** Comparison of tan delta viscoelastic ratio for cervical, thoracic and lumbar spinal cord. Error bars represent standard deviation. Exact *p* values are listed in Supplementary Table S6.

For all three anatomical areas (cervical, thoracic, and lumbar spinal cord tissues), the compound, storage, and loss moduli responded similarly to increasing strain rates (Figures 7A–C). Notably, the complex modulus values of ex vivo spinal cord tissue rose nonlinearly with rising test frequency (Figure 7D). Additionally, at every strain rate, it was shown that the tissue in the thoracic spinal cord was significantly more rigid than the tissue in the cervical or lumbar spinal cord, with compound modulus values roughly 1.5-times that of the lumbar region (Figure 7D). There was no statistically significant variation in terms of the compound modulus, between the cervical and lumbar sections of the spinal cord (*p* > 0.05; Figure 7D; Supplementary Table S6). Despite higher compound modulus values for spinal cord for various test frequencies between 0.05 and 1.00 Hz, they rose more quickly at applied rates between 0.05 and 0.50 Hz, as indicated by a steeper gradient as test frequency increased (Figure 7D). This led researchers to conclude that the perceived stiffness of spinal cord was highly dependent on strain rate; however, there was no significant difference between the three anatomical locations in the rate of change. Between 0.50 and 1.00 Hz, however, spinal cord tissue exhibited limited further stress-stiffening activity, with further slow rises approaching to a plateau (thoracic region) or further slight decreases (cervical and lumbar regions) in modulus. A limited ability for additional stiffening above 0.50 Hz demonstrated that all sites of the spinal cord share some inherent mechanical characteristics.

At strain rates between 0.05 and 0.50 Hz, tan δ values for thoracic (that is, 0.26, 0.25, 0.06, respectively) and lumbar (that is, 0.27, 0.25, 0.07, respectively) spinal cord regions were similar, respectively, which were higher than cervical (that is, 0.21, 0.21, 0.04, respectively) region. However, beyond 0.50 Hz, the tan δ readings of the cervical spinal cord started to grow much more than those of the thoracic and lumbar spinal cords (Figure 7E; Supplementary Table S6). At 1.00 Hz, the cervical spinal cord’s tan δ value was 0.769, whereas the thoracic and lumbar spinal cords merely reached 0.656 and 0.638, respectively. The cervical region had significantly lower tan δ values than the lumbar and thoracic regions, demonstrating a differential viscoelastic reaction relying on local anatomical position.

## 4 Discussion

Dependent on dynamic mechanical analyses at the mesoscopic level, this work carried out a series of measurements to comprehensively characterize regional mechanical properties differences of spinal cord tissue via measures of elasticity, energetics, and viscoelasticity. The mechanical response of spinal cord tissues was shown to be highly nonlinear and viscoelastic, with strong hysteretic behavior and rate dependency. The results emphasized the importance of dynamic mechanical analyses in investigating the regional specific differences in spinal cord tissues to better understand the biomechanical properties of the spinal cord. To our knowledge, this is the first study to investigate region-specific differences in spinal cord tissue using dynamic indentation testing and dynamic mechanical analyses. These findings may provide a unique quantitative framework for refining/adjustment of existing biomechanical models, and offer new perspectives for regenerative medicine applications.

Significant conditioning effects were observed in cervical, thoracic, and lumbar spinal cord tissues *ex vivo*. In this study, these effects could be restored to the “unconditioned” response after at least 5 min of re-equilibration between tests. The findings are aligned with the inherent properties exhibited by porcine brain tissue when evaluated *in vivo*, *in situ* and *in vitro* in uniaxial indentation (Prevost et al., 2011a). These conditioning effects may be a result of interstitial water diffusion in the tissue (Prevost et al., 2011b). CNS tissue are considered as highly hydrated. Estimations of standard hydration levels in spinal cord tissue suggest that fluid comprises roughly 80% of the mass of the spinal cord. Though interstitial fluid diffusion was not explicitly included in the previous brain model by Prevost et al. (2011a), the proposed formulation for the bulk response was based on a simplified qualitative analysis of the role of hydrating fluid in determining the apparent volumetric tissue behavior. Briefly, the undeformed tissue volume is divided into an incompressible component and a compressible component. This partition mirrors a separation of the hydrating fluid in a “bound” component, which does not diffuse freely under loading, and an “interstitial” component, which diffuses in response to hydrostatic loading, accommodating volumetric tissue deformation. The bulk mechanical response of biological materials exhibited a significant increase in stiffness at higher rates of deformation, attributed to a notable decrease in interstitial fluid diffusion. In addition, the current study revealed that the degree of conditioning investigated was rate-dependent, with larger conditioning effects observed at higher rate. This might suggest that using other methods such as confined techniques to limit fluid loss could be important.

It was discovered that the mechanical characteristics of the spinal cord tissues vary greatly by anatomical location. The thoracic spinal cord was noticeably more rigid than the cervical and lumbar spinal cords. Recent research by Bartlett et al. (2020) suggests that the thoracic spinal cord could react differently to external strain than the cervical or lumbar spinal cord when displacement is evaluated over time. Due to the unique loading configurations, differences in indentation responses between three anatomical locations may be enhanced in the present study, despite the fact that the indenter radius for the present study was still within the basic guidance advised by Garo et al. (2007) (about 10% of the specimen thickness). Regarding the reasons why such disparities may occur, there is no agreement. Several researches (LaRocca, 1988; van Dommelen et al., 2010; Matsunaga and Sakou, 2012; Koser et al., 2015; Baumann et al., 2020; Cooper et al., 2020) hypothesized a significant correlation between the ultrastructure and mechanical characteristics of spinal cord tissues. Cell types, sizes, morphologies, and locations, as well as the composition of the extracellular matrix, could change significantly between various areas of the central nervous system, and may alter during development, aging, and pathological circumstances. Actually, a better knowledge of the complexities of tissue activity *ex vivo* is essential for the development of reliable spinal cord models based on mesoscopic tissue quantification data. Additionally, it was discovered that the viscoelastic characteristics of spinal cord tissue differ by anatomical area. The tan δ readings suggested that the cervical spinal cord responds in a variety of ways to lower strain rates than the thoracic or lumbar spinal cord, with the cervical spinal cord showing a greater inclination for elasticity instead of viscous behavior. The elastic property of an item is its resistance to external deformation. This discovery may indicate self-regulation/adaptation and may explain why chronic extradural pressing of cervical spinal cord (e.g., cervical spondylotic myelopathy, ossification of the posterior longitudinal ligament, etc.) may not end up severe symptoms in the short term (LaRocca, 1988; Matsunaga and Sakou, 2012).

Tissue from the spinal cord behaved nonlinearly to escalating strain rates, demonstrating viscoelastic characteristics comparable to those previously observed (Shetye et al., 2014; Budday et al., 2017). We believe that rapid identification of spinal cord tissue following dissection is necessary to guarantee that measured mechanical parameters accurately reflect genuine values, as prior findings have indicated that mechanical properties differ significantly with inclining post-mortem time (Oakland et al., 2006; Bartlett et al., 2016). The findings of Oakland et al. (2006) declared that the tangent modulus, which is measured by the slope of a stress-strain curve when the strain is kept constant, can increase with the passage of time. This has substantial ramifications, in particular when spinal cord tissue is stored for longer durations before being subjected to mechanical testing and the influence of various storage settings and temperatures is uncertain. In addition, this testing technique did not involve any significant tissue preparation or preconditioning. Preconditioning is a possible cause of external variance (Morgan et al., 2014). The number of preconditioning cycles has the potential to mislead and alter spinal cord tissue end modulus measurements, according to empirical research (Oakland et al., 2006; Cheng et al., 2009). While it is widely acknowledged that preconditioning aids in minimizing inter-sample variability, a uniform procedure for doing preconditioning stays controversial. High inter-sample variability is caused by the high moisture content of native spinal cord tissue; nevertheless, attempts to minimize the inter-sample variability should avoid intentionally modifying tissue to the point that it loses its inherent properties. Extensive preconditioning, for instance, has been demonstrated to affect the orientation of collagen fibers in other anisotropic tissues, such as ligaments (Quinn and Winkelstein, 2011). Therefore, researchers must recognize that preconditioning could alter tissue architecture and confuse final findings (Hosseini et al., 2014).

Additionally, the main aim of this work was to examine the viscoelastic of spinal cord tissue characteristics under moderate strain rates (range from 0.05 to 1.00 Hz). Multiple researches have been conducted up to this point to quantify modulus at high strain rate values. Although it is anticipated that they are likely to be valuable for the construction of computerized SCI models, they are probably less useful in the context to tissue engineering. Due to the fact that tissues in the spinal cord could be subjected to certain strains during non-pathological activities, future study should strive to record the mechanical characteristics of spinal tissue while it is experiencing normal physiological movement.

Soft tissues are not perfectly elastic materials or homogeneous, and they typically display both viscous and elastic properties that are dependent on time. Unfortunately, reported values of elastic modulus for a given soft tissue can span several orders of magnitude. Part of the variation in reported elastic modulus values stems from variation in controllable experimental variables. Examples include *in vivo versus ex vivo* measurements, sample preparation, sample size, time from death/tissue excision, temperature, storage medium, testing method, and tissue microstructure. These experimental differences make direct comparison of results between studies difficult. Discrepancies in spinal cord tissue mechanics may be attributed to differences in the methodology, including injury type, sample preparation (fresh, thick sections versus thin, freeze-thawed samples that promote ice crystal mediated tissue damage). Moreover, variations in reported modulus values for tissues may stem from the application of different elastic models. When consistent raw data points are achieved from tests, it is sometimes necessary to fit a modelling equation to the raw data in order to extract some tissue properties. Such models used improperly or inconsistently may add to the variability in elastic modulus. Collectively, we think that soft biological tissues (such as spinal cord) may not have a single elastic modulus value independent of experimental method, and that modulus values for a single tissue can span several orders of magnitude.

Identifying relevant mechanical and biochemical factors and correlating nervous tissue stiffness to microstructure are important open questions with immediate applications in neuromechanics and neuroprotection. The elastic moduli of central CNS reported in the literature range between ∼50 and 20,000 Pa (Franze et al., 2013). Both apparent discrepancies may be explained by the highly complex, nonlinear, viscoelastic properties of CNS tissue; time and length scales of the mechanical tests significantly influence the results (McKee et al., 2011; Franze et al., 2013). Cell types, sizes, morphologies, and distributions, as well as the extracellular matrix (ECM) components may vary significantly between different regions in the spinal cord tissue, and they may change during development and ageing as well as in pathological conditions. Koser et al. (2015) makes a first attempt to propose to correlate cell number density, myelin content, collagen content, ECM composition, and axonal orientation, with the calculated apparent Young’s moduli of the spinal cord tissue regions. Unfortunately, in central nervous tissue, these correlations are far less one-toone as they are in other hard and soft tissues. For example, we know that bone stiffness is strongly correlated with density and arterial stiffness increases with collagen content.

Biocompatibility and tissue-matching of implanted biomaterials is a major consideration in tissue engineering, particularly when translation from the lab to the clinic is the primary aim. Hydrogel implantation offers multiple benefits for SCI therapy. Hydrogels form a substitute extracellular matrix and porous structure which modifies the injury environment promoting cellular and axon infiltration (Perale et al., 2011; Haggerty et al., 2017). Hydrogels can be used as a vehicle for molecular (Hyatt et al., 2010), drug (Perale et al., 2012) or cell-based ‘combinatorial’ therapies (Macaya et al., 2013; Führmann et al., 2016). A recent study by Jon Prager et al. (2020) showed that the stiffness of hydrogels encapsulating a clinically relevant transplant population (olfactory ensheathing cells) can be measured by ultrasound elastography, enabling synthesis of hydrogels with comparable stiffness to canine spinal cord injury. The authors demonstrated proof-of-principle of a novel approach to stiffness-matching hydrogel-olfactory ensheathing cell implants to ‘real-life’ SCI values; an approach applicable to multiple biomaterial implants for regenerative therapies. In another study (Bartlett et al., 2020), the authors described a bespoke mechanical characterisation method that facilitated robust measurement of fresh spinal cord and brain tissue and allowed direct like-for-like mechanical benchmarking for matching clinical-grade hydrogels suitable for regenerative medicine. They reported differences in the mechanical properties of spinal cord tissue, and quantified the extent of mechanical anisotropy within the cervical spinal cord. They then demonstrated that the mechanical properties of clinical-grade collagen, fibrin and alginate hydrogels can be tuned to closely mimic the mechanical properties of different regions within the CNS. We believe that this present work will serve as a basis for future studies to link spinal cytoarchitecture before and after injury to tissue mechanical characteristics with the ultimate goal of tuning spinal cord tissue mechanics toward successful functional repair also in humans.

We evaluated adult rat spinal cord tissue *ex vivo*, which limits our physiological understanding of the tissue. Owing to the supersoft character of spinal cord tissue, even under optimal transport and storage settings, it is possible that changes in the mechanical characteristics of new *ex vivo* tissue could occur if the perfusion pressure decreased (Metz et al., 1970; Etz et al., 2009). This work may have been restricted by the fact that *ex vivo* analyses did not precisely simulate *in vivo* settings, though the measurement methods were widely used to compile the majority of the data pertaining to the mechanical characteristics of spinal cord tissues (Fiford and Bilston, 2005; Clarke et al., 2009). However, this should not have impacted a comparison of the relative mechanical behaviors of different regional spinal cord tissue. Besides, translation of rat cord properties to human tissue has not yet been demonstrated. However, it is impossible, clinically and ethically, to obtain fresh (within 2 h of death), normal, intact human spinal cord tissue. Rat cord may not be the best surrogate though the rat spinal cord model has been considered, by an increasing published data, as an appropriate model to study for investigating SCI mechanisms (Kjell and Olson, 2016; García et al., 2019; Speidel et al., 2020). In the absence of human tissue measures, such mechanical characterization aids the improvement of animal preclinical models and would help the development of tissue engineering for spinal cord transplants. The need for biomechanical experiments using larger animals such as pigs may be considered in the near future. Finally, the indentation technique used could only measure a single uniaxial force-displacement. Considering the local spinal cord tissue behaved like an anisotropic material under indentation (Bartlett et al., 2020), complementary multiple-axial methods around the indentation’s locus may supply beneficial extra information to the tissue mechanical characteristics in the future.

## 5 Conclusion

This work reported the nonlinear region-, rate-, and time-dependent mechanical properties of regional dynamic mechanical response of spinal cord tissue to *ex vivo* indentation, and discuss potential relationships between these structures and its potential function. On specimens of adult rat spinal cord, we acquired experimental data from a variety of dynamic loading modes (indentation). We successfully described the loading-mode specific regional (i.e., cervical, thoracic, and lumbar) behavior of the spinal cord tissues and highlighted the complicated characteristics intrinsic to the tissue response (hysteresis, rate dependence, non-linearity and conditioning effects). As such, the comprehensive mechanical characterization of spinal cord, as provided in this study, lays a foundation for further comparison between healthy and diseased spinal cord to the future development of spinal cord scaffold and helps to advance our knowledge of neuroscience.

## Data Availability

The original contributions presented in the study are included in the article/Supplementary Material, further inquiries can be directed to the corresponding authors.
