# Supplementary material for: Regional biomechanical characterization of the spinal cord tissue: dynamic mechanical response
Source: Front Bioeng Biotechnol. 2024 Aug 16;12:1439323. doi: 10.3389/fbioe.2024.1439323 (PMC11361947; doi:10.3389/fbioe.2024.1439323)
Supplement: Supplementary file 1 [file Table1.DOCX]

**Supplementary Tables**

**Supplementary Table 1.** **Peak force for representative sets of measurements performed for cervical, thoracic and lumbar spinal cord tissues at four different rates.**

| Segment | Frequency (Hz) | Dura on (mN) | Dura off (mN) | *P* value |
| --- | --- | --- | --- | --- |
| Cervical spinal cord | **0.05** | 9.21 ± 2.16 | 1.59 ± 0.41 | < 0.001 |
|  | **0.10** | 8.50 ± 1.09 | 2.09 ± 0.68 | < 0.001 |
|  | **0.50** | 10.23 ± 0.82 | 3.08 ± 1.01 | < 0.001 |
|  | **1.00** | 12.22 ± 0.55 | 3.39 ± 0.57 | < 0.001 |
| Thoracic spinal cord | **0.05** | 8.40 ± 1.03 | 1.68 ± 0.10 | < 0.001 |
|  | **0.10** | 8.36 ± 0.49 | 2.03 ± 0.56 | < 0.001 |
|  | **0.50** | 10.28 ± 1.02 | 4.03 ± 0.38 | < 0.001 |
|  | **1.00** | 10.85 ± 1.09 | 4.40 ± 0.67 | < 0.001 |
| Lumbar spinal cord | **0.05** | 7.08 ± 0.75 | 1.08 ± 0.23 | < 0.001 |
|  | **0.10** | 10.58 ± 1.39 | 1.40 ± 0.06 | < 0.001 |
|  | **0.50** | 10.89 ± 0.54 | 2.27 ± 0.38 | < 0.001 |
|  | **1.00** | 12.22 ± 0.55 | 2.63 ± 0.30 | < 0.001 |

Data are presented as average ± standard deviation. *P* values were obtained using independent samples *t*-test. This table corresponds to Figure 2B-D.

**Supplementary Table 2.** **Statistical comparison of the peak force reached at the end of the first loading ramp ex vivo under five conditions.**

| Segment |  | II | III | IV | V |
| --- | --- | --- | --- | --- | --- |
| Cervical spinal cord | **I** | 0.263 | 0.114 | 0.781 | 1.000 |
|  | **II** |  | 0.994 | 0.924 | 0.252 |
|  | **III** |  |  | 0.735 | 0.103 |
|  | **IV** |  |  |  | 0.789 |
| Thoracic spinal cord | **I** | 0.935 | 0.347 | 0.853 | 0.988 |
|  | **II** |  | 0.819 | 1.000 | 0.998 |
|  | **III** |  |  | 0.897 | 0.650 |
|  | **IV** |  |  |  | 0.987 |
| Lumbar spinal cord | **I** | 0.994 | 0.446 | 0.967 | 0.982 |
|  | **II** |  | 0.307 | 0.894 | 0.901 |
|  | **III** |  |  | 0.957 | 0.771 |
|  | **IV** |  |  |  | 0.999 |

For cervical and thoracic spinal cord tissues, the differences among different velocities were tested by a one-way ANOVA with Scheffe’s *post hoc* analysis to obtain the multiple comparison *P* values. For lumbar spinal cord tissue, the differences among different velocities were tested by a Kruskal-Wallis ANOVA followed by Games-Howell’s *post hoc* analysis to obtain the multiple comparison *P* values. This table corresponds to Figure 4.

**Supplementary Table 3.** **Statistical comparison of the peak forces reached at the end of the first loading ramp and of the second loading ramp for the four different rates of indentation.**

| Frequency (Hz) |  | Cervical spinal cord (mN) | Thoracic spinal cord (mN) | Lumbar spinal cord (mN) |
| --- | --- | --- | --- | --- |
| 0.05 | **Unconditioned** | 1.51 ± 0.55 | 1.84 ± 0.49 | 1.04 ± 0.28 |
|  | **Conditioned** | 0.74 ± 0.36 | 0.94 ± 0.39 | 0.48 ± 0.23 |
|  | ***P* value** | < 0.001 | < 0.001 | < 0.001 |
| 0.10 | **Unconditioned** | 1.99 ± 0.70 | 2.61 ± 0.64 | 1.44 ± 0.58 |
|  | **Conditioned** | 1.31 ± 0.56 | 1.71 ± 0.48 | 0.86 ± 0.42 |
|  | ***P* value** | 0.004 | < 0.001 | 0.002 |
| 0.50 | **Unconditioned** | 3.24 ± 0.72 | 3.97 ± 1.12 | 2.35 ± 0.97 |
|  | **Conditioned** | 2.70 ± 0.59 | 3.17 ± 0.88 | 1.90 ± 0.74 |
|  | ***P* value** | 0.022 | 0.027 | 0.133 |
| 1.00 | **Unconditioned** | 3.27 ± 0.70 | 4.73 ± 1.30 | 2.42 ± 0.79 |
|  | **Conditioned** | 2.84 ± 0.57 | 3.88 ± 0.93 | 2.09 ± 0.73 |
|  | ***P* value** | 0.053 | 0.034 | 0.214 |

Data are presented as average ± standard deviation. *P* values were obtained using independent samples *t*-test. This table corresponds to Figure 5E.

**Supplementary Table 4.** **Statistical comparison of the conditioning ratios for each indentation rate for ex vivo spinal cord tissue.**

| Segment |  | 0.10 Hz | 0.50 Hz | 1.00 Hz |
| --- | --- | --- | --- | --- |
| Cervical spinal cord | **0.05 Hz** | < 0.001 | < 0.001 | < 0.001 |
|  | **0.10 Hz** |  | < 0.001 | < 0.001 |
|  | **0.50 Hz** |  |  | 0.001 |
| Thoracic spinal cord | **0.05 Hz** | < 0.001 | < 0.001 | < 0.001 |
|  | **0.10 Hz** |  | < 0.001 | < 0.001 |
|  | **0.50 Hz** |  |  | 0.056 |
| Lumbar spinal cord | **0.05 Hz** | 0.027 | < 0.001 | < 0.001 |
|  | **0.10 Hz** |  | < 0.001 | < 0.001 |
|  | **0.50 Hz** |  |  | 0.050 |

The differences among various rates were tested by a Kruskal-Wallis ANOVA with Games-Howell’s *post hoc* analysis to obtain the multiple comparison *P* values. This table corresponds to Figure 6.

**Supplementary Table 5. Statistical comparison of the conditioning ratios for ex vivo spinal cord tissue at specific indentation rates.**

|  | 0.05 Hz | | 0.10 Hz | | 0.50 Hz | | 1.00 Hz | |
| --- | --- | --- | --- | --- | --- | --- | --- | --- |
|  | Thoracic | Lumbar | Thoracic | Lumbar | Thoracic | Lumbar | Thoracic | Lumbar |
| Cervical | 0.763 | 0.706 | 0.976 | 0.271 | < 0.001 | 0.166 | 0.001 | 0.612 |
| Thoracic |  | 0.299 |  | 0.226 |  | 0.744 |  | 0.116 |

Regarding the rate of 0.05 Hz, the differences among different velocities were tested by a one-way ANOVA with Turkey’s *post hoc* analysis to obtain the multiple comparison *P* values. In terms of other rates (i.e., 0.10, 0.50 and 1.00 Hz), the differences among various rates were tested by a Kruskal-Wallis ANOVA with Games-Howell’s *post hoc* analysis to obtain the multiple comparison *P* values. This table corresponds to Figure 6.

**Supplementary Table 6.** **Statistical comparison of the compound modulus and tan delta for ex vivo spinal cord tissue at different indentation rates.**

| Segment | Variables | | | | | | | |
| --- | --- | --- | --- | --- | --- | --- | --- | --- |
|  | **Compound modulus comparison** | | | | | | | |
|  | **0.05 Hz** | | **0.10 Hz** | | **0.50 Hz** | | **1.00 Hz** | |
|  | Thoracic | Lumbar | Thoracic | Lumbar | Thoracic | Lumbar | Thoracic | Lumbar |
| Cervical | 0.509 | 0.073 | 0.138 | 0.419 | 0.603 | 0.053 | 0.030 | 0.103 |
| Thoracic |  | 0.004 |  | 0.007 |  | 0.004 |  | 0.001 |
|  | **Tan delta comparison** | | | | | | | |
|  | **0.05 Hz** | | **0.10 Hz** | | **0.50 Hz** | | **1.00 Hz** | |
|  | Thoracic | Lumbar | Thoracic | Lumbar | Thoracic | Lumbar | Thoracic | Lumbar |
| Cervical | 0.023 | 0.008 | 0.002 | 0.002 | 0.127 | 0.040 | 0.292 | < 0.001 |
| Thoracic |  | 0.923 |  | 0.989 |  | 0.859 |  | < 0.001 |

Regarding compound modulus, the differences among various rates were tested by a Kruskal-Wallis ANOVA with Games-Howell’s *post hoc* analysis to obtain the multiple comparison *P* values for 1.00 Hz, and the differences among different velocities were tested by a one-way ANOVA with Turkey’s *post hoc* analysis to obtain the multiple comparison *P* values for 0.05, 0.10 and 0.50 Hz. In terms of tan delta, the differences among different velocities were tested by a one-way ANOVA with Turkey’s *post hoc* analysis to obtain the multiple comparison *P* values. This table corresponds to Figure 7D-E.
